# A new species of *Monstrilla* (Copepoda, Monstrilloida) from the plankton of a large coastal system of the northwestern Caribbean with a key to species

**DOI:** 10.3897/zookeys.876.38400

**Published:** 2019-09-25

**Authors:** Eduardo Suárez-Morales, Iván A. Castellanos-Osorio

**Affiliations:** 1 El Colegio de la Frontera Sur (ECOSUR), Av. Centenario Km. 5.5, A.P. 424, Chetumal, Quintana Roo77014, Mexico El Colegio de la Frontera Sur Chetumal Mexico

**Keywords:** estuaries, crustaceans, parasitic copepods, taxonomy, tropical zooplankton

## Abstract

The genus *Monstrilla* Dana, 1849 is the most diverse of the copepod order Monstrilloida. Monstrilloid copepods are endoparasites of benthic polychaetes and molluscs; adult individuals are free-living, non-feeding reproductive forms that briefly become part of the zooplankton community, where they are occasionally captured by plankton nets. Monstrilloid copepods are frequently found during routine plankton samplings of coastal and estuarine habitats, but they are rarely found in large numbers. The western sector of the Caribbean Sea is known to harbor a diverse monstrilloid fauna. The analysis of zooplankton samples obtained during nine years from Chetumal Bay, a large embayment of the Mexican Caribbean coast, yielded a male monstrilloid that was found to represent a new species. It is herein described following upgraded standards and compared with its congeners. A key to males and females of the *Monstrilla* species known from the northwestern Caribbean is also provided.

## Introduction

Monstrilloid copepods are protelean parasites of benthic invertebrates; most juvenile stages are endoparasitic and adult individuals are free-living, non-feeding reproductive forms that briefly become part of the zooplankton community, where they are occasionally captured by plankton nets (Suárez-Morales 2011). As parasites they cause a strong inflammatory response in its hosts (Suárez-Morales et al. 2010). Because of their rarity in the plankton and taxonomic complexity, there are large geographic areas in which the monstrilloid copepod fauna remains largely unknown (Suárez-Morales 2011, 2015). According to Suárez-Morales (2011), the regions with the highest number of monstrilloid records are the North Atlantic (32 species), followed by the northwestern Caribbean Sea and the Gulf of Mexico (24), the region around Indonesia, Malaysia, the Philippines, and Japan (20+), the Mediterranean-Black Sea region (19), and the Brazilian-Argentine coasts (16). In the Caribbean Sea, most records, particularly of the genus *Monstrilla* Dana, 1849, are from its westernmost area, the Mexican Caribbean coast (Suárez-Morales and Gasca 1992; Suárez-Morales 1994, 1995, 1996, 1998, 2003).

At more than 2500 km^2^, Chetumal Bay is the largest estuarine lagoonal system of the Mexican Caribbean coast. It is a priority protection area for the conservation of the Caribbean manatee, both nationally and internationally (Morales-Vela et al. 2003).

A large set of 607 zooplankton samples was obtained over a period of nine years (1990–1997, 2015, 2016). Different zooplankton groups have been studied in Chetumal Bay, including medusae (Suárez-Morales and Segura-Puertas 1995), appendicularians and chaetognaths (Gasca and Castellanos 1993), fish and crustacean larvae (Gasca et al. 1994), planktonic copepods (Ruíz-Pineda et al. 2016), as well as zooplankton biomass variations (Vásquez-Yeomans et al. 2012). Despite these intense sampling efforts, no monstrilloid copepods have previously been obtained in this lagoonal system. During a zooplankton haul performed in July 1997 at station 12, an adult male specimen of the genus *Monstrilla* was collected. After its taxonomic analysis, this monstrilloid was found to represent a new species which is herein described following upgraded standards (Grygier and Ohtsuka 1995, 2008) and compared with its known congeners. A key to the species of *Monstrilla* known from the Mexican Caribbean is also provided.

## Material and methods

**Figure 1.**
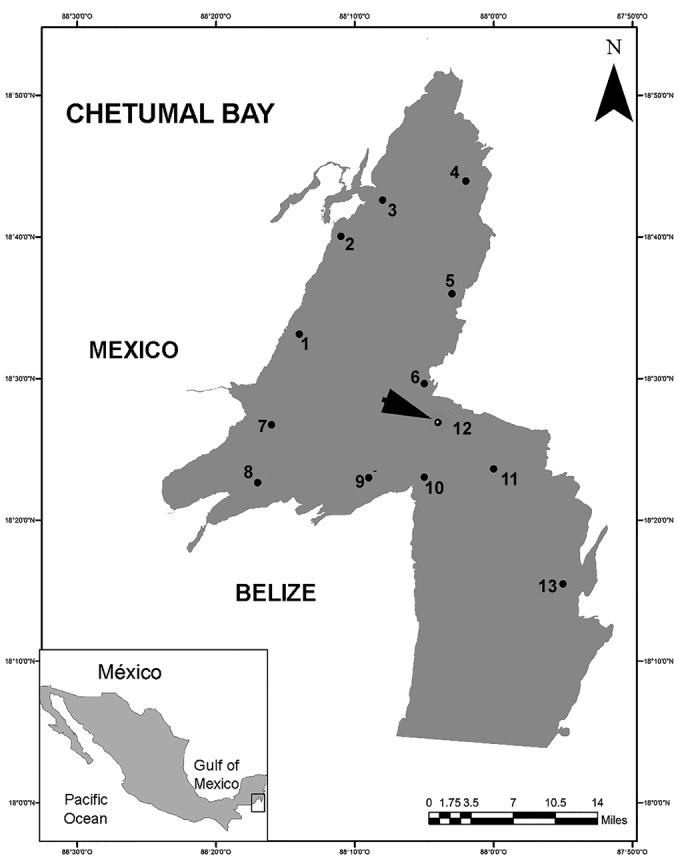
Surveyed area in Chetumal Bay showing zooplankton sampling sites.

Zooplankton samples were obtained monthly in 1997 by performing daytime surface trawls at each of 13 sampling stations in Chetumal Bay on the southern coast of the Mexican Caribbean (Fig. 1). A standard 1.2 m long plankton net was used having a 0.45 m diameter mouth and 0.1 mm filtering mesh. The volume of filtered water was estimated with a digital flowmeter. The filtered volume values fluctuated between 132 and 232 m^3^.

The new species is herein described in full following the current upgraded descriptive standards in monstrilloid taxonomy (Grygier and Ohtsuka 1995, 2008). The morphologic terminology follows Huys and Boxshall (1991). The holotype specimen was deposited in the collection of zooplankton held at El Colegio de la Frontera Sur (ECOSUR), Chetumal, Mexico (ECO-CHZ), where it is available for consultation.

## Taxonomy

### Subclass Copepoda Milne-Edwards, 1840

**Order Monstrilloida Dana, 1849**

**Family Monstrillidae Dana, 1849**

**Genus *Monstrilla* Dana, 1849**

#### Monstrilla chetumalensis sp. nov.

Taxon classificationAnimaliaMonstrilloidaMonstrillidae

DDC7875C-526E-52B6-8D86-F21DE274EA89

http://zoobank.org/87550E34-4F19-465A-BBDF-42BD18CF4C1D

**Figure 2.**
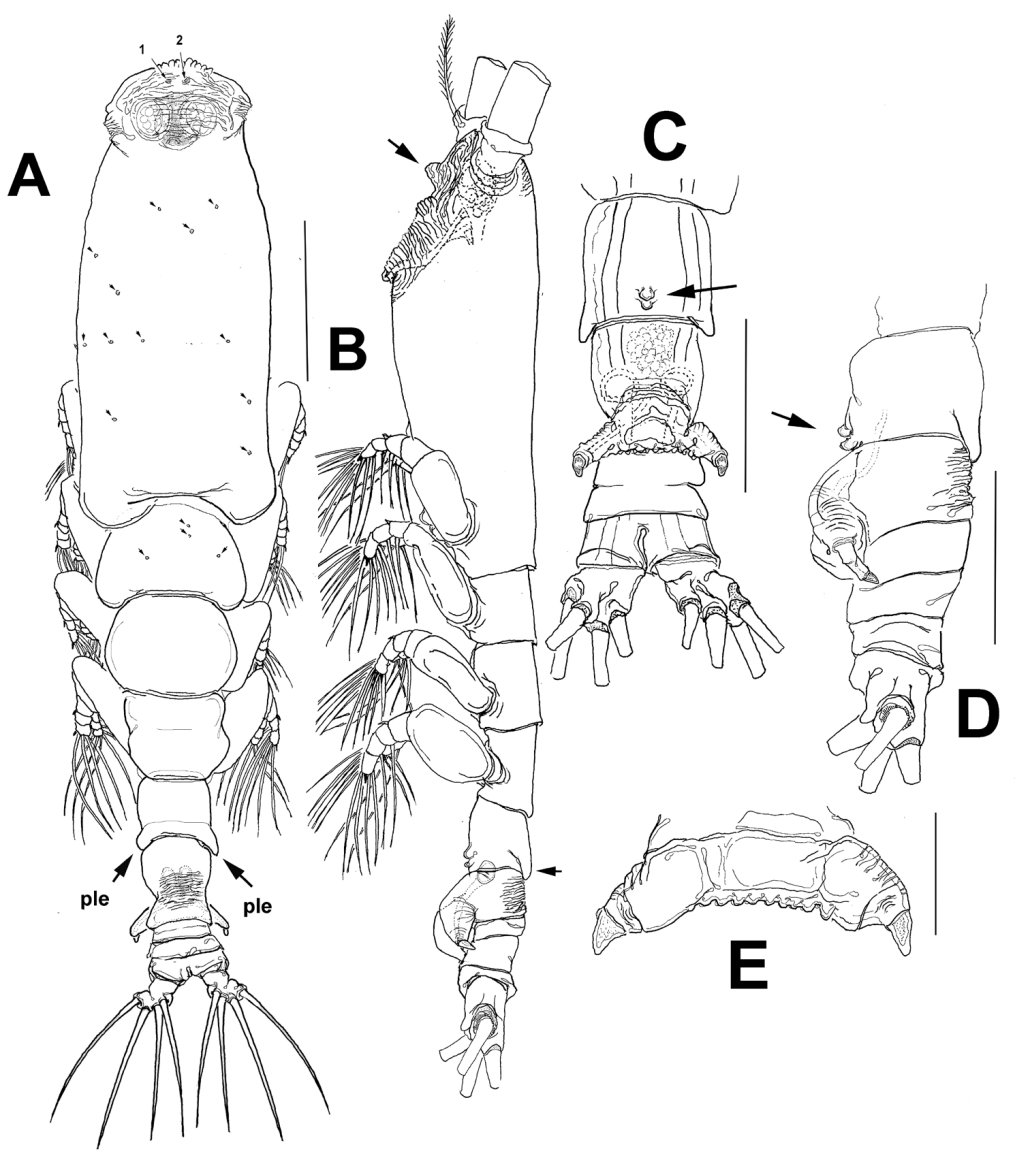
*Monstrilla chetumalensis* sp. nov., male holotype **A** habitus dorsal view **B** habitus lateral view, arrow indicates medial ventral protuberance **C** urosome ventral view; arrow indicates ventral globular processes on fifth pedigerous somite **D** urosome lateral view; arrow indicates ventral globular processes on fifth pedigerous somite **E** genital complex with lappets, ventral view. Scale bars: 100 μm. (**A–C**)

**Figure 3.**
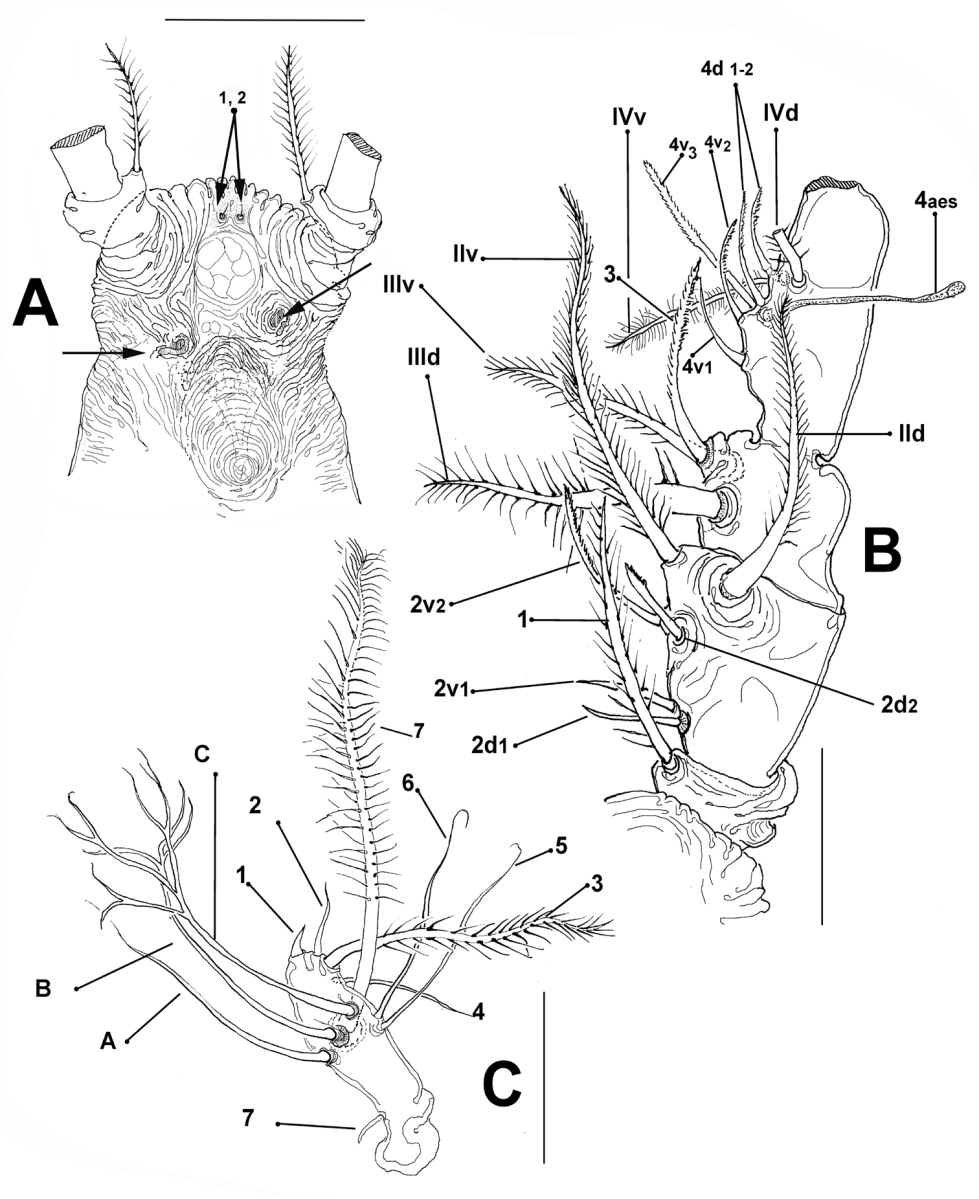
*Monstrilla chetumalensis* sp. nov., male holotype (**A–E, C**) **A** anterior part of cephalosome ventral view; arrow indicates nipple-like processes; s = sensilla **B** antennule segments 1–4 in dorsal view showing setal elements (sensu Grygier and Ohtsuka 1995) **C** fifth antennulary segment with setal elements (sensu Huys et al. 2007) **D** urosome lateral view **E** genital complex with lappets ventral view. Scale bars: 200 μm (**A, B**), 100 μm (**C, D**), 50 μm (**E**).

**Figure 4.**
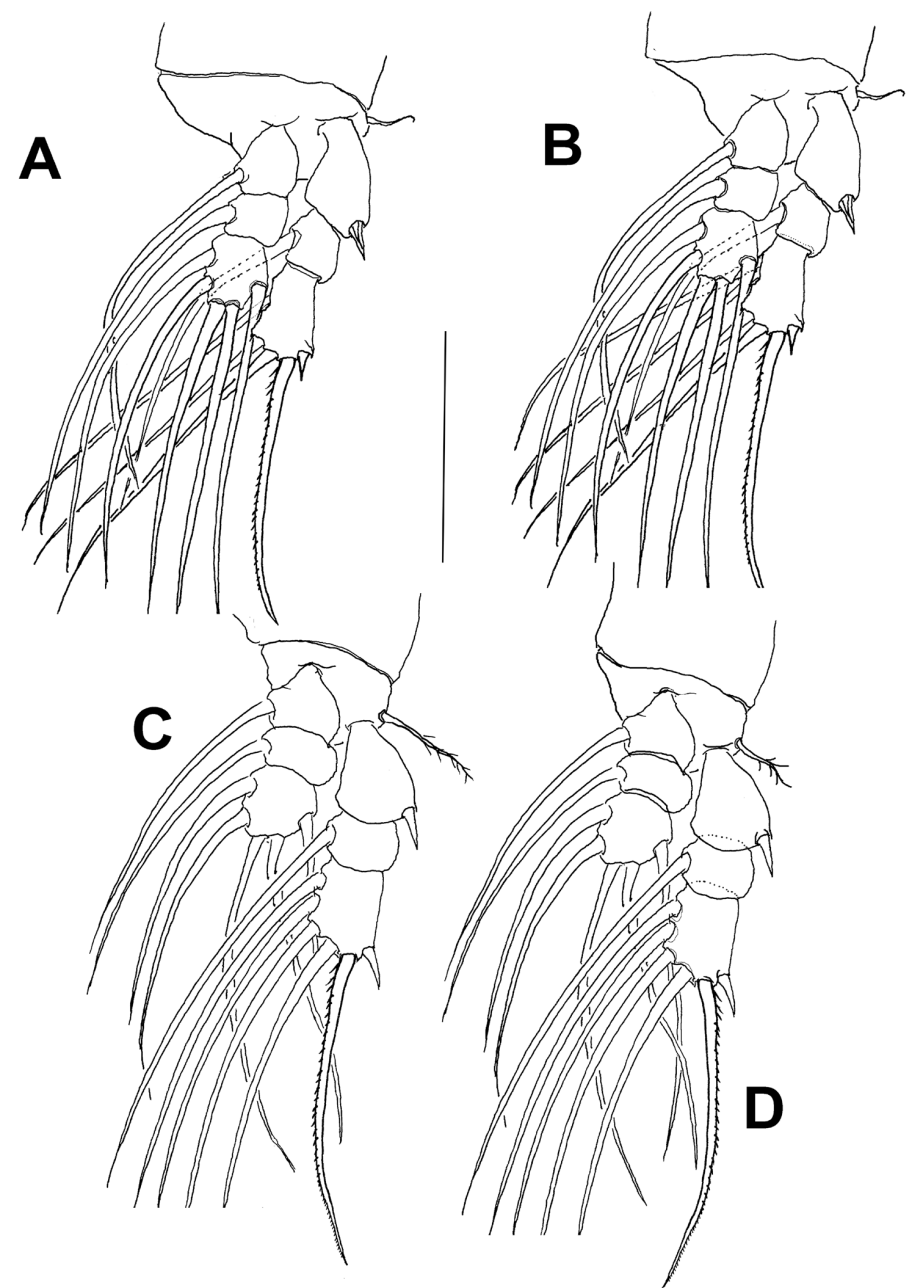
*Monstrilla chetumalensis* sp. nov., male holotype **A** leg 1 **B** leg 2 **C** leg 3 **D** leg 4. Scale bars: 100 μm.

Figures 2 , 3 , 4 , Table 1

##### Material examined.

Holotype adult male (ECO-CH-Z-10330), Chetumal Bay, near Mexico-Belize international border (18°26'54"N; 88°04'00"W) on 27 July 1997 by I. Castellanos-Osorio. Specimen partially dissected, cephalothorax and urosome in a vial, ethanol-preserved. Appendages including antennules and legs 1–4 mounted on semi-permanent slide with glycerine, sealed with acrylic varnish.

##### Etymology.

The epithet of the new species is a toponym that refers to Chetumal Bay, the type locality of this species.

##### Diagnosis.

Small-sized male *Monstrilla* (0.73 mm), with body divided in relatively short, robust prosome, pedigerous somites 2–4 tapering posteriorly, and slender urosome. Cephalothorax with low, rounded medial rostral projection, with both dorsal and ventral cuticular ornamentation. Antennule 5-segmented geniculate antennules. Geniculation between segments 4 and 5. Fifth pedigerous somite separated from preceding somite. Posterolateral margins produced and partially overlapping succeeding somite, visible in lateral and dorsal views. Somite with two small rounded ventral processes visible in lateral view. Legs 1–4 with outer sea on basis; exopods and endopods 3-segmented. Leg 5 absent. Genital somite with dorsal field of transverse striations; ventral genital complex represented by short shaft with distal laterally diverging lappets with rugose anterior surface, branches with dorsally directed apical spiniform processes, probably representing opercular flaps; lappets connected medially by dentate margin. Caudal rami with four subequally long caudal setae.

##### Description of adult male holotype.

Body shape and tagmosis as usual in male *Monstrilla* (Huys and Boxshall 1991; Suárez-Morales 1993, 1996, 2003) (Fig. 2A, B). Total body length of holotype individual 0.69 mm, measured from anterior end of cephalothorax to posterior margin of anal somite. Cephalothorax representing 47.5% of total body length. Succeeding pedigerous somites 2–4 each with pair of biramous swimming legs; pedigerous somites 2–4 combined accounting for 31% of total body length in dorsal view. Cephalic region wide, bilaterally protuberant in dorsal view, narrower than cephalothorax; outer margin of cephalic protuberances corrugate. Pair of small dorsal pit setae present between antennulary bases; ventral anterior surface also with two pit setae (1, 2 in Figs 2A, 3A, respectively). Forehead moderately produced, weakly rounded, with coarsely rugose anterior margin and field of transverse striations on dorsal anterior surface; no other cephalic ornamentation discernible on dorsal anterior surface (Fig. 2A). Cephalothorax robust, 0.36 mm long, representing 47.5% of total body length; dorsal surface with scattered dorsal pores (Fig. 2A). Midventral oral papilla moderately protuberant (Fig. 2B), located at about proximal 1/3 (0.31) along ventral surface of cephalothorax. Pair of relatively small lateral pigment cups moderately developed, separated by length of less than one eye diameter, weakly pigmented; ventral cup slightly larger than lateral cups. Preoral ventral surface with low, wide-based rounded process protruding between antennulary bases (arrowed in Fig. 2B); nipple-like cuticular processes surrounded by striated surface (Fig. 3A).

**Table 1.** Armature of legs 1–4 including basis, exopods, and endopods. (Roman numerals indicate spiniform elements, Arabic numbers indicate setiform elements, set from inner to outer positions).

	**Basis**	**Exopod**	**Endopod**
Leg 1	1-0	0–I;0–1;2,2,I	1-0;1-0;2,2,I
Legs 2–4	1-0	0–I ;1-0;2,2,1, I	1-0;1-0; 2,2,1, I

Antennule relatively robust (Fig. 3B), 5-segmented; length = 0.53 mm, representing 38% of total body length, and 75% of cephalothorax length, with segments 1–4 separated by complete sutures. Intersegmental division between segments 3 and 4 lacking suture, division marked by constriction; segment 4 being longest: geniculation between segments 4 and 5 (Fig. 3B). Armature, using terminology of Grygier and Ohtsuka (1995) for female monstrilloid antennular armature of segments 1–4 complemented with nomenclature by Huys et al. (2007) for elements on male fifth antennule segment, antennulary segment (1) element 1 present on first; element setiform, setulated, distinctively long, reaching well beyond distal margin of second segment. (2) elements 2d_1, 2_, 2v_1–3_, and IId expressed on second segment. (3) third segment with elements 3, IIId, and IIIv with setal element 3 setiform, pinnate, remarkably long, reaching beyond proximal half of succeeding fourth segment. (4) Segment four bearing normally developed elements 4d_1, 2_ and 4v_1–3_ as well as setae IVd, IVm, and IVv; elements of group 4v_1–3_ short, spiniform, except for long, setiform, spinulose element 4v_3_. Slender aesthetasc 4aes in ventral position. (5) terminal segment armed as follows (sensu Huys et al. 2007): elements 1–7 present on anterior margin, with three branched setal elements A–C (Fig. 3C); segment with small apical aesthetasc (element 2 in Fig. 3C). Terminal segment lacking unusual features or ornamentation (Fig. 3C).

Legs 1–4 with smooth intercoxal sclerites of rectangular, smooth. Bases with straight inner margins; outer basal setal sparsely setulose on legs 1–4; on leg 3, outer basal seta about twice as long as and slightly thicker than in other legs. Endopods and exopods of triarticulated, outer margins of exopods smooth. All elements setiform and biserially plumose except for outer spines on first segments and outer apical spiniform seta on third exopodal segments displaying and third exopodal segments displaying sparsely spinulose inner margin and smooth outer margin (Fig. 4A–D). Armature of legs 1–4 as:

Urosome consisting of fifth pedigerous, genital somite (carrying genital complex), two short, free postgenital somites divided by incomplete dorsal suture, and short anal somite (Fig. 2B–D). Fifth pedigerous somite with ventrally produced proximal half; dorsal surface smooth. Distal half of fifth pedigerous somite with pair of small medial rounded processes visible in lateral view (arrowed in Fig. 2C, D). Posterolateral margins of fifth pedigerous somite produced, partially overlapping succeeding genital somite both dorsally and laterally (“ple” in Fig. 2A, arrow in Fig. 2B). Genital somite slightly shorter than fifth pedigerous somite; genital complex of type I (Suárez-Morales and McKinnon 2014), represented by short, robust ventrally expanded shaft; complex with short, widely divergent lappets tapering distally into apical subtriangular opercular process (Fig. 2C–E). Lappets with rugose anterior surface, branches connected medially by wide dentate margin. Anal somite 1.3 times as long as genital somite. Caudal rami subquadrate, approximately 1.1 times as long as wide and about 0.7 times as long as anal somite. Each ramus armed with four subequally long caudal setae (Fig. 2A).

##### Remarks.

The new species differs from the males of other known congeners in several respects. Firstly, there are only a few other male *Monstrilla* with divergent genital lappets that point backwards and end in a subtriangular or spiniform opercular process. The male of *Monstrilla chetumalensis* sp. nov. most closely resembles the Indian species *Monstrilla lata* Desai & Bal, 1962. Both have similar body proportions, cephalothorax ornamentation, and paired, divergent genital lappets, each with a short distal opercular structure. However, in *M. chetumalensis*, the lappets are strongly curved and have an inverted U-shape (Desai and Bal 1962, figs 4, 5). Moreover, the antennulary armature differs strongly between these two species, particularly in the size and number of setal elements of segments 1–4 (Desai and Bal 1962: fig.3). In addition, *M. lata* has six caudal setae (Desai and Bal 1962: fig. 5) vs four in the new species (Fig. 2A). In *M. lata* the fifth pedigerous somite has a weak concavity in its proximal half (Desai and Bal 1962: fig.4), whereas the same structure is ventrally produced in *M. chetumalensis* (Fig. 2D). The genital complex of the new species shares some features with *M. papilliremis* Isaac, 1975 from South Africa. Both have divergent lappets with distal subtriangular opercular structures pointing backwards (Isaac 1975); however, in *M. papilliremis*, the lappets are medially connected by a smooth margin with a medial notch and also have an inverted U-shape, thus diverging from the conditions observed in the genital complex of *M. chetumalensis*. In two other well-known species of the genus, *M. longicornis* Thompson, 1890 and *M. longiremis* Giesbrecht, 1893, the genital lappets are also divergent, connected medially by a smooth, straight margin (Sars 1921; Huys and Boxshall 1991; Suárez-Morales 2010) and thus diverging from the dentate condition observed in *M. chetumalensis* sp. nov. In addition, both *M. longicornis* and *M. longiremis* have a 1-segmented leg 5 (see Sars 1921; Suárez-Morales 2010), which is absent in the new species. Backwardly directed genital lappets, as those observed in *M. chetumalensis* sp. nov., were reported in male *M. longicornis* by Huys and Boxshall (1991) and also in male *M. longiremis* by Suárez-Morales (2010). Secondly, the presence of small rounded ventral processes on the fifth pedigerous somite have been reported previously only in the Caribbean *M. marioi* Suárez-Morales, 2003 (Suárez-Morales 2003: fig. 4), but in this species the process involves three small lobes (Suárez-Morales 2003: fig. 4) instead of two observed in *M. chetumalensis* sp. nov. (Fig. 2D). The distinctive characters observed in our male specimen appear to be enough evidence to support its assignment as a new species.

Also, we considered the resemblance of the described male with males of the recently described genus *Caromiobenella* Jeon, Lee & Soh, 2018, which is known from males only. It has been recognized (Grygier and Ohtsuka 2008) that the type species of *Monstrilla*, *M. viridis* Dana, 1849 should be redescribed from a neotype in order to clearly define the genus delimitation and clarify the status of related genera. Morphological comparison shows that *M. chetumalensis* sp. nov. is assignable to *Monstrilla*, as it clearly diverges from *Caromiobenella* in the following characters:

In male *Caromiobenella* branched antennulary setae are absent from the fifth antennulary segment (Jeon et al. 2018: fig. 2C), but in the new species setae A and B of thee fifth antennulary segment are branched (Fig. 3C). The cephalothoracic ornamentation in *Caromiobenella* spp. includes two pairs of large dorsal crater-like depressions and pitted sensilla (Jeon et al. 2018: fig. 1A); these structures are absent in *M. chetumalensis* in which the cephalothoracic ornamentation is represented mostly by a conspicuous pattern of striations on its ventral surface and a few scattered dorsal pores (Fig. 2A, B). In addition, the two outermost setae on the third exopodal segments of legs 1–4 are serrate along the outer margin and smooth along the inner margin in *Caromiobenella* (Jeon et al. 2018: fig. 3C–E). In *M. chetumalensis* a distinct condition was observed: these exopodal setae are either smooth or serrate along the inner margin (Fig. 4A–D). Also, species of *Caromiobenella* have five or six caudal setae (Jeon et al. 2018), whereas only four caudal seta are present in the new species (Fig. 2A). According to Jeon et al. (2018), the presence of a type 3 male antennule (see Huys and Boxshall 1991; Suárez-Morales 2011), with a modified fifth segment bearing distal transverse serrate ridges on the inner distal margin, is a diagnostic character to species of *Caromiobenella*. The new species has an unmodified (type I) antennule. In *Caromiobenella* the genital complex is represented by a robust shaft and short, subtriangular non-divergent distal lappets with a medial smooth protrusion (Jeon et al. 2018: fig.7C) and paired medial opercular flaps. In *M. chetumalensis* the genital complex is also robust and short, but lappets are strongly divergent and are medially joined by a dentate margin. No such medial opercular flaps were observed in the new species; these are probably represented by the terminal structures on the tip of each lappet.

Currently, the are eight species of *Monstrilla* recorded from different coastal or reef areas of the Mexican Caribbean: *M. reidae* Suárez-Morales, 1993a (male) from Bahia de la Ascensión, *M. mariaeugeniae* Suárez-Morales and Islas-Landeros, 1993 (female) from off Puerto Morelos reef zone, *M. ciqroi* (Suárez-Morals, 1993b) (female) from Bahia de la Ascension, *M. barbata* Suárez-Morales & Gasca, 1992 (female) (see Suárez-Morales et al. 2006), *M. elongata* Suárez-Morales, 1994 (both sexes) (see Suárez-Morales 1996) from Puerto Morelos reef zone, *M. globosa* Suárez-Morales, 2003 (male) and *M. marioi* Suárez-Morales, 2003 (male), and *M. rebis* Suárez-Morales, 1993b (female) from Bahia de la Ascension (see Suárez-Morales and Gasca 1992; Suárez-Morales 1993a, 1993b, 1996, 2003; Suárez-Morales and Islas-Landeros 1993; Suárez-Morales et al. 2006).

### Key to the female *Monstrilla* of the Mexican Caribbean

**Table:** 

1	Antennules indistinctly segmented, slender or robust	**4**
–	Antennules distinctly 4-segmented	**2**
2	With irregularly rugose medial rostral process	***M. barbata* Suárez-Morales & Gasca, 1992**
–	Medial rostral process absent	**3**
3	A Fifth leg with small rounded protuberance adjacent to inner lobe	***M. ciqroi* (Suárez-Morales, 1993b)** *
–	Fifth leg with inner margin of fifth leg smooth	***M. rebis* Suárez-Morales, 1993b**
4	Antennule with straight anterior and posterior margins; fifth leg with 1 lobe armed with 2 setae, inner margin smooth	***M. elongata* Suárez-Morales, 1994**
–	Antennule with rounded protuberances along anterior and posterior margins; fifth leg with 1 lobe armed with 2 setae and with strong spiniform process on inner margin	***M. mariaeugeniae* Suárez-Morales and Islas-Landeros, 1993**

### Key to male *Monstrilla* of the Mexican Caribbean

**Table:** 

1	Fifth legs absent	**2**
–	Fifth legs present, with 1 lobe armed with single seta	***M. elongata* Suárez-Morales, 1994** **
2	Genital complex with lappets directed backwards; fifth pedigerous somite with two small globular processes	***M. chetumalensis* sp. nov.**
–	Genital complex with lappets not directed backwards	**3**
3	Genital complex with inverted U-shaped lappets tapering into acute points	***M. marioi* Suárez-Morales, 2003**
–	Genital complex with different structure, elongate, cylindrical, rod-like, with paired globular processes in terminal position	**4**
4	Fifth pedigerous somite with short digitiform ventral process; apical antennulary element 6_1_ bifurcate	***M. globosa* Suárez-Morales, 2003**
–	Fifth pedigerous somite lacking ventral process; apical antennulary element 6_1_ not bifurcate	***M. reidae* Suárez-Morales, 1993a**

## Supplementary Material

XML Treatment for Monstrilla chetumalensis

## Footnotes

* originally described as Monstrillopsis; assigned to Monstrilla by Suárez-Morales et al. (2006).

** Male described as M. wandelii tropica by Suárez-Morales (1996)
